# Genetically Engineered Cellular Membrane Vesicles as Tailorable Shells for Therapeutics

**DOI:** 10.1002/advs.202100460

**Published:** 2021-09-08

**Authors:** En Ren, Chao Liu, Peng Lv, Junqing Wang, Gang Liu

**Affiliations:** ^1^ State Key Laboratory of Molecular Vaccinology and Molecular Diagnostics & Center for Molecular Imaging and Translational Medicine School of Public Health Xiamen University Xiamen 361102 China; ^2^ School of Pharmaceutical Sciences (Shenzhen) Sun Yat‐sen University Guangzhou 510275 China

**Keywords:** cellular membrane vesicles, genetically engineered tactics, surface modification, therapeutics carriers

## Abstract

Benefiting from the blooming interaction of nanotechnology and biotechnology, biosynthetic cellular membrane vesicles (Bio‐MVs) have shown superior characteristics for therapeutic transportation because of their hydrophilic cavity and hydrophobic bilayer structure, as well as their inherent biocompatibility and negligible immunogenicity. These excellent cell‐like features with specific functional protein expression on the surface can invoke their remarkable ability for Bio‐MVs based recombinant protein therapy to facilitate the advanced synergy in poly‐therapy. To date, various tactics have been developed for Bio‐MVs surface modification with functional proteins through hydrophobic insertion or multivalent electrostatic interactions. While the Bio‐MVs grow through genetically engineering strategies can maintain binding specificity, sort orders, and lead to strict information about artificial proteins in a facile and sustainable way. In this progress report, the most current technology of Bio‐MVs is discussed, with an emphasis on their multi‐functionalities as “tailorable shells” for delivering bio‐functional moieties and therapeutic entities. The most notable success and challenges via genetically engineered tactics to achieve the new generation of Bio‐MVs are highlighted. Besides, future perspectives of Bio‐MVs in novel bio‐nanotherapy are provided.

## Graphical abstract

The functional proteins can be anchored on the cell surface via genetic engineering tactics for various application prospects including cancer immunotherapy, vaccine delivery, virus/bacterial toxin capture, cancer oncolytic virotherapy, and chemotherapy drugs delivery.

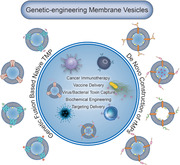

## 1 Introduction

The central dogma in medicine is to steer therapeutic loads to target tissues and cellsfor achieving maximal efficacy with minimal toxic effects.^[^ ^1^ ^]^ Recently, a comprehensive strategy that imitates the design principle and mechanism of “guided missiles” that require three fundamental components involving the recognition moieties, drug carriers, and appropriate linkers is paving the way for drug delivery. These components should meet criteria including efficient drug loading, systemically non‐toxic, and blood circulation stability to improve the delivery efficiency.^[^ ^2^ ^]^ While straightforward in principle, most of these traits usually come at the expense of one of the others, and it has so far been impossible to combine all traits into a nano‐platform.^[^ ^3^ ^]^ The fate of synthesized “missiles” depends upon the different components that can overcome these complexities to achieve full benefits from each component.

**Scheme 1:**
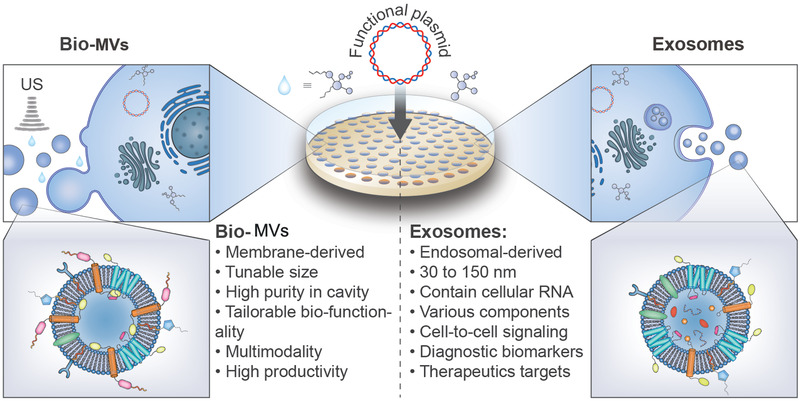
The procedure for purification and preparation of biosynthetic cellular membrane vesicles (Bio‐MVs) and exosomes. A) The prepared Bio‐MVs are usually generated with the addition of surfactants and ultrasonication to control their size distribution. Benefiting from the complete ultrasonic crushing of primary cells and following a centrifugal separation, Bio‐MVs can minimize the aqueous impurities (e.g., enzymes, oligonucleotides) in the cavity and have great significance in exogenous substances transportation.^[^ ^7^, ^42^ ^]^ B) Exosomes are secreted from all cell types when the multi‐vesicular bodies (MVBs) fuse with the cytoplasmic membrane. The size distribution of exosomes is ≈30–150 nm in diameter and plays an integral role as intercellular communication vectors because of many soluble proteins and an array of oligonucleotides in the lumen. Besides, they are always regarded as diagnostic biomarkers and therapeutic targets.^[^ ^8^, ^9^ ^]^

Considering these conundrums, there has been a growing interest in utilizing biologic nanocarriers with membranous structures as synthetic “missiles” to develop innovative therapeutics.^[^ ^4^ ^]^ Their chief advantages include the native cavity structure for drug loading, diverse modification tactics for membrane surface, and naturally negligible immunogenicity in blood circulation.^[^ ^5^ ^]^ Among numerous membranous nanocarriers, the biosynthetic cellular membrane vesicles (Bio‐MVs) produced from the natural cell membrane have emerged as a promising alternative for biotherapy.^[^ ^6^ ^]^ The inspiration for Bio‐MVs comes from the hijacking host cell machinery of enveloped viruses and the subsequent release (budding process) from the infected cells.^[^ ^7^ ^]^ Once the original cells are treated by certain amounts of surfactants and ultrasonic crushing, cell membrane fragments will undergo a reassembly process to form tunable Bio‐MVs (the particle size can be controlled from 50 to 2500 nm in diameter).^[^ ^6^, ^7^ ^]^ (**Scheme 1**A) As is well known to all, the exosomes (30–150 nm in diameter) are naturally secreted from all cell types and bodily fluids, which are enclosed by a phospholipid membrane bilayer.^[^ ^8^ ^]^ Exosomes are generated in multi‐vesicular bodies (MVBs) and secreted when MVBs fuse with the cytoplasmic membrane.^[^ ^9^ ^]^ (Scheme 1B) In the process, not only some specific transmembrane proteins (TMPs) like CD9, CD63, CD81, CD82 were anchored as a specific marker; the lumen of exosomes also possesses many biological contents, such as soluble enzymes, mitochondrial DNA, mRNA, microRNA (miRNA), or other functional genes.^[^ ^10^ ^]^ Benefiting from the characteristics that the secretion of exosomes is closely related to various physiological processes such as inflammation, homeostasis, and autoimmune diseases or cancer, the identification of pathological exosomes has been regarded as diagnostic biomarkers.^[^ ^6^, ^11^ ^]^ Their inherent merits to transfer cargos have improved their possibility of being used to deliver a variety of agents, including chemotherapeutics, miRNAs, siRNAs, proteins, and even nanoparticles.^[^ ^6^, ^10^, ^12^ ^]^ Nevertheless, the inherent substances in the lumen have their unique function that may increase the complexity of components for therapeutic application.^[^ ^5^, ^13^ ^]^ Compared with the secreted exosomes, the Bio‐MVs possess similar morphological structures, physicochemical properties, and membrane protein components but contain relatively smaller amounts of cellular RNA and other soluble proteins.^[^ ^7^ ^]^ These characteristics were assessed by the western blot analysis for plasma membrane‐protein (Na^+^/K^+^‐ATPase), anti‐cell nucleus (Histone H3a) antibody, and anti‐cytoplasm protein such as actin and glyceraldehyde‐3‐phosphate dehydrogenase (GAPDH) antibody.^[^ ^7^ ^]^ Specifically, the Bio‐MVs produced from the reassembled cell membrane fraction exhibit high productivity to meet the clinical needs.^[^ ^14^ ^]^ Therefore, the Bio‐MVs may provide an alternative choice as “missiles” for exogenous substances or functional moieties delivery.

On account of the efficient and facile procedure of separation and purification together with the various cell types (e.g., red blood cells (RBCs), T lymphocytes, natural killer (NK) cells, macrophage) in the biological world, the Bio‐MVs endowed with a wide variety of preexisting functionalities.^[^ ^15^ ^]^ Their pharmacokinetics and therapeutic efficiency strongly depend on the membrane protein composition, and the diversity of expressed membrane proteins will have a decisive influence on their function.^[^ ^16^ ^]^ Therefore, many synthetic biological approaches have been developed to artificially control TMP expression and diversity for satisfying clinical requirements entirely.^[^ ^17^ ^]^ The strategy most worth applauding is the genetic decorating procedure for Bio‐MVs modification with desired proteins on the surface.^[^ ^17a^ ^]^ Versus the traditional modification methods such as multivalent electrostatic interactions, receptor‐ligand binding, and hydrophobic insertion, this strategy successfully hijack the cellular activity and has several unique advantages: 1) the artificial proteins/peptides/domains can avoid undesired protein orientation, structural distortions and interfere with the existed membrane proteins because of cellular strict genetic control expression system; 2) the integrated proteins anchoring in the Bio‐MVs by genetic engineering could have more post‐translational modifications (e.g., glycosylation, acetylation, phosphorylation) that are critically important for their biological functions; 3) the existence of degeneracy and usage bias for genetic codes has excellent benefits for the same functional proteins expressing in different species but maintain their original sequence information and functions.^[^ ^17^, ^18^ ^]^ With their inherent hollow structure as an ideal cargo carrier for other therapeutics encapsulated inside, Bio‐MVs will be a portfolio “missile” for medical treatment.^[^ ^5^, ^19^ ^]^ The resulting Bio‐MVs are one kind of ideal “tailorable shell” for promoting the delivery of functional cargos and other functional proteins.

**Scheme 2:**
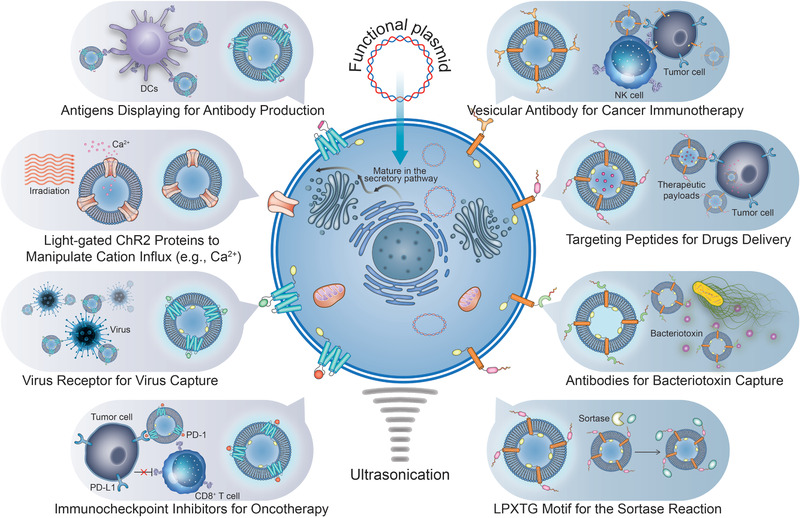
Schematic illustration of the design philosophy, the preparation process of genetically engineered Bio‐MVs. Benefiting from the genetic engineering tactics, the artificial proteins (antibody, antigen, virus epitope, iron channel proteins, functional peptides, recognition receptors, etc.) can be modified on the cell membrane with specificity, sort orders, and strict information of themselves maintaining. Following prepared Bio‐MVs with these specific customize proteins anchoring on surface, just like “tailorable shells” with interior space for other moieties encapsulation, will have great potential for cancer immunotherapy, vaccine delivery, virus/bacterial toxin capture, cancer oncolytic virotherapy as well as cancer theranostics.

In this progress report, we highlight genetically engineered Bio‐MVs based “missile” biosynthesis and their multi‐functionalities as “tailorable shells” to deliver functional moieties, including the small molecular cargos and various proteins. (**Scheme** **2**) A brief introduction of genetic‐engineering methods for Bio‐MVs surface modification will be given first. Then varieties of representative functionalities will have a detailed introduction from standard functional peptides to abundant complex proteins, including antibodies and other specific membrane receptors. The mechanisms and synergistic effects of encapsulated functional moieties with engineered Bio‐MVs will have a detailed description. In the end, we will discuss the historical context of this specific technology and evaluate the complexities, potential pitfalls, and opportunities presented by different functional proteins anchoring.

## 2 Design Philosophy of Genetically Engineered Bio‐MVs

The theoretical basis of a genetic‐engineering method for Bio‐MVs is an in‐depth understanding of the information and their corresponding structures of membrane proteins.^[^ ^20^ ^]^ In principle, TMPs are amphipathic and allow them to contact extracellular water conditions and the phospholipid bilayer of cell membranes.^[^ ^21^ ^]^ That is the central issue for their heterologous expression and purification in a soluble state.^[^ ^22^ ^]^ Generally, TMPs share two necessary compartments for cell membrane anchoring: the cleavable N‐terminal signal sequences and hydrophobic peptide regions.^[^ ^23^ ^]^ The signal sequence acts as a zip code to dominate the targeting to and translocation across the endoplasmic reticulum, localization in the Golgi apparatus, and finally transported to the cytoplasmic membrane.^[^ ^24^ ^]^ These specific hydrophobic‐rich peptides will play significant roles in protein displaying on the cell membrane surface of particular importance here. A TMP directly spans the membrane depends on the alternating series of signal sequences and the number of transmembrane regions.^[^ ^25^ ^]^ It is imperative that all TMP features, including structure characteristics, hydrophilic and hydrophobic peculiarity, and bio‐physiological property, are ubiquitous to all prokaryotes and eukaryotes.^[^ ^26^ ^]^ Besides, degeneracy and usage bias for genetic codes have excellent benefits for the same functional proteins expressing in different species but maintain their original sequence information and functions. For example, the light‐gated channelrhodopsins‐2 proteins (ChR2) from the *C. reinhardtii* and vesicular stomatitis virus G‐protein (VSVG) from the virus could be expressed on the HEK293T cells or cancer cells and correctly maintain their intrinsic functions simultaneously, such as regulating the calcium ion transporting and membrane fusion.^[^ ^16^, ^27^ ^]^ Other than above mentioned native TMPs, most functional peptides screed in vitro, functional protein domains, and secretory proteins are incapable of anchoring on the cell membrane. This section will focus on the modification strategies for these proteins expressing on the cell membrane surface. To date, two approaches have been developed for membrane engineering based on the protein sorting mechanisms mentioned above.

### 2.1 Genetic Fusion Based Native TMPs

Membrane proteins are the most crucial component for the maintenance of cell homeostasis and viability.^[^ ^28^ ^]^ They have critical roles in signaling and adhesion as well as recognition of molecules or recipient cells.^[^ ^16^, ^29^ ^]^ Bioinformatics on TMPs offers insight into artificial control of the expression process. One sensible strategy will be a genetic fusion‐based native TMPs enriched in original cell membranes to ensure optimum localization of expressed products. This strategy requires a solid understanding of the location of signal peptide sequences, transmembrane region sequences, and topological domains to native TMPs.^[^ ^21^, ^24^, ^26^ ^]^ The information can guide the right insertion site for the exogenous amino acid sequence. The desired amino acid sequences can be integrated into any desired destination sites of targeted proteins benefiting from the breakthroughs of gene editing methods.^[^ ^30^ ^]^ For example, Kell and glycophorin A (GPA) are two kinds of membrane proteins with an exposed C terminus and N terminus on the mature RBCs with a single transmembrane region. Based on the structural information, the LPXTG motif for the sortase A reaction allows stable integration on the outer leaflet of the cell membrane in vitro via the genetic engineering method.^[^ ^31^ ^]^ These artificially expressed motifs could maintain their property and introduce a single‐domain antibody attached to the cell membrane with a sortase A reaction.^[^ ^32^ ^]^ Another model anchoring protein is the Lamp2b and lipid‐binding domain C1C2 of MFGE8 ( = lactadherin).^[^ ^33^ ^]^ The Lamp2b is a membrane protein found abundantly in exosomal membranes.^[^ ^34^ ^]^ With the neuron‐specific specific rabies viral glycoprotein (RVG) peptide fusing to the N terminus, the purified RVG‐targeted vesicles can deliver siRNA specifically to neurons, microglia, oligodendrocytes in the brain, resulting in a specific gene knockdown.^[^ ^34a^ ^]^ Besides, when the OVA antigen fusing with the C1C2 and prepared OVA‐C1C2‐encoding, the antigen‐specific CD4^+^ T lymphocyte induction could be specifically favored.

Apart from those proteins with a single transmembrane region or domain, large complex proteins with multiple transmembrane regions are also appropriate targeting proteins for modification. For example, Zachary et al. selected complicated tetraspanins as a modification objective that transversed the cell membrane four times. Based on the accurate serial sequence information, they successfully constructed a set of recombinant fluorescent proteins for both the inner and outer surfaces displaying at different selected tetraspanin sites.^[^ ^16a^ ^]^ Moreover, other membrane proteins with several trans‐membrane hydrophobic sequences like Rab5a or lactadherin could be used for luciferase expression either.^[^ ^33^ ^]^

### 2.2 De Novo Construction of Recombinant Membrane Proteins

The sequence information of various secretory proteins and peptides screened in vitro is strictly required for their functions like the targeting peptide and specific antibody.^[^ ^1^, ^35^ ^]^ These specific functional moieties cannot offer membrane anchoring because of their inherent amino acid sequences and intrinsic biological functions. Though the sensible strategy of TMPs based genetic fusion mentioned above is an effective method, the exogenous sequence insertion still has the risk of affecting the original proteins. For instance, the selected target protein may be a cytotoxic factor that maintains normal cell growth, and random sequence insertion may break the intracellular homeostasis for intricated signaling pathways.^[^ ^36^ ^]^ Besides, some complex proteins are difficult to engineer into intrinsic TMPs because of their stringent restrictions of disulfide bonds for their biological function.^[^ ^37^ ^]^ Hence, an ingenious design tactic of *de novo* construction of recombinant membrane proteins (rMPs) was developed.^[^ ^7^ ^]^ Basically, varieties of recombinant membrane proteins could be reconstructed when a signal peptide sequence and a transmembrane region sequence are together genetically fusing to the targeted domains. After these functional components (recombined gene expression sequences) are expressed in the target cells together, this kind of recombinant protein will target the location from the cytoplasm to the cell plasma membrane based on the sorting mechanism to their TMPs.^[^ ^38^ ^]^ As an alternative method for TMPs, several advantages are apparent: 1) It has almost no influence on the original proteins and sequence information because the N‐terminal signal sequences will be cleaved by signal peptidase; 2) all pieces of information, such as structural bio‐physiological characteristics on the target proteins and sequence identity, are maintained, which guaranteed their functional perfection; 3) the customized proteins will artificially display on the outside or inside of the cell membrane according to requirements; 4) several functional proteins or domains can be collaboratively displayed for synergistic effects in principle; 5) the membrane proteins can be artificially customized through hijacking the intrinsic membrane protein expression mechanisms and then the cellular protein component.^[^ ^7^, ^38^, ^39^ ^]^ Combined with the diversities of functional cells, there is every valid reason to believe the successful establishment of rMPs strategy can greatly improve the Bio‐MVs performance.

## 3 Multi‐Functionalities of Genetically Engineered Bio‐MVs

To date, various kinds of functions for Bio‐MVs have been identified based on their biochemistry, including their compositions and biological roles.^[^ ^5^, ^40^ ^]^ For example, with intrinsic protein expression and nucleic acids encapsulation, these Bio‐MVs can be applied for antigen presentation, intercellular communication, and other pathological roles.^[^ ^41^ ^]^ Apart from their peculiarities, other functions with Bio‐MVs surface modification tactics have developed either including specific targeting moieties' linkage, expression of exogenous proteins, and other chemical receptors anchoring.^[^ ^42^ ^]^ On account of the particular cavity structure, Bio‐MVs can be used for chemotherapeutics delivery, fluorescent molecules loading, and immunotherapy for a spectrum of diseases, including cancers, infections, and immune disorders.^[^ ^42b,c^ ^]^ Of the most significant concern, both the external surface and internal cavity structure of Bio‐MVs will be the independent functional moieties anchoring platform, which constitutes an excellent carrier for synergistic therapeutic drugs.^[^ ^40^ ^]^ Herein in this section, we focus on the additional functions based on the exogenous proteins anchoring via genetic engineering methods. Various functionalities will have a detailed introduction, from simple amino acid sequences like functional peptides to complex proteins such as antibodies, specific cellular receptors, and viral envelope proteins.

### 3.1 Functional Peptides Engineering

Functional peptides are usually screened in vitro through phage displaying technology and have been widely used in biomedical applications because of their specific targeting ability, desirable pharmacokinetics, low immunogenicity, and toxicity.^[^ ^43^ ^]^ Despite these particular characteristics, native peptides are seldom directly used for nanomedicine because they may lose their bioactivity before reaching the intended lesions under enzymatic degradation and renal clearance.^[^ ^44^ ^]^ Till now, varieties of strategies have been developed to enhance metabolic stability, prolong blood circulation, and increase the binding affinity of peptides. For example, the PEGylation, the dimeric or multimeric peptide system, and even cyclization of the peptide have been introduced to overcome these problems.^[^ ^44^ ^]^ On the other hand, the targeting peptides are usually decorated with nanocarriers (e.g., liposomes, metal‐organic frameworks (MOFs), biomacromolecules, superparamagnetic iron oxide nanoparticles (SPION)) via chemical decoration methods for other functional moieties delivery. Moreover, parts of these peptides have been selected as various pathogen substitutes for prophylactic vaccines.^[^ ^45^ ^]^ In the process, appropriate methods that bridging them with other synergistic moieties are daunting challenges because the strict information of functional peptides should maintain strictly for their functionality.^[^ ^46^ ^]^ This part will introduce the genetically engineered Bio‐MVs as “tailorable shells” for functional peptides anchoring, including the tumor‐targeting peptides and virus peptide epitope. Benefiting from their exact information anchoring on the Bio‐MVs and characteristics of natural drug carriers, the cancer‐targeting ability and efficiency of antigen delivery have vast improvement.

#### 3.1.1 Bio‐MVs Based Adenovirus Camouflaging for Oncolytic Virotherapy

As an effective new antitumor method, oncolytic virotherapy that utilizes oncolytic adenovirus (OA) to selectively induce cell necrosis or apoptosis has shown promising results in preclinical studies and clinical trials.^[^ ^47^ ^]^ Owing to their ability to self‐replicate within the cancer cells, the oncolytic virus has unique pharmacokinetics distinct from conventional therapeutics.^[^ ^48^ ^]^ However, varieties of concerns that have emerged from clinical trials are the intrinsic identity of antivirus immunity, innate immune response, and poor targeting delivery of systemically administered OA.^[^ ^49^ ^]^ Despite these daunting challenges, major strides have been made, such as expressing pro‐drug convertases and cytokines for specific tumor targeting, but therapy in patients with intact immunity will remain a significant challenge.^[^ ^50^ ^]^ Taking inspiration from nanotechnology is an alternative approach to shield viruses chemically with polymers such as polyethylene glycol or poly‐(N‐(2‐hydroxypropyl) methacrylamide) (pHPMA).^[^ ^51^ ^]^ These hydrophilic polymers can be chemically cross‐linked on viruses to shield them from preexisting antibody responses and reduce the new antibody and T‐cell responses. However, this resulted in reduced targeting efficacy and attenuated therapeutic efficacy due to the steric hindrance introduced by those macromolecular polymers.^[^ ^51^ ^]^ Therefore, the desired modification strategy to achieve robust antiviral immune shielding and unique targeting capability for oncolytic virotherapy is urgently needed.

**Figure 1:**
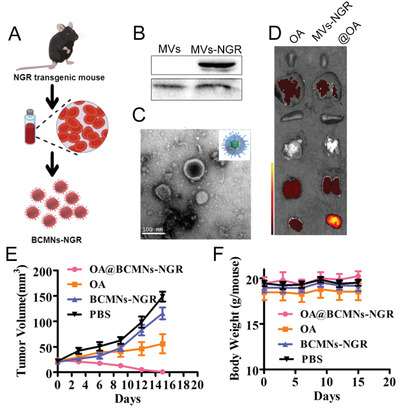
Genetic‐engineering biosynthetic cellular membrane vesicles (Bio‐MVs) with NGR tripeptide (Asn‐Gly‐Arg) displaying for oncolytic adenovirus (OA) delivery. A) Schematic illustration for the synthetic process. B) Western blotting analysis to detect the NGR peptide and marker protein Alix on the Bio‐MVs. C) Transmission electron microscopy (TEM) images of purified OA@Bio‐MVs which were negatively stained with uranyl acetate. Scale bar: 100 nm. D) Fluorescence images of different tissues and organs after injected with OA and NGR‐MVs@OA. E) Tumor growth curves and body weight of tumor bearing nude mice after various kinds of OA treatments. Reproduced with permission.^[^ ^52^ ^]^ Copyright 2019, American Chemical Society.

Considering the excellent performance of Bio‐MVs as functional moieties encapsulation, Lv et al. synthesized a new kind of Bio‐MVs‐based “guided shells” for OA camouflaging and delivery.^[^ ^52^ ^]^ Herein, the NGR tripeptide (Asn‐Gly‐Arg) as the targeting ligand was selected for Bio‐MVs modification because the NGR could recognize a membrane‐bound metalloproteinase (APN) of tumor cells.^[^ ^53^ ^]^ (**Figure** **1**) First, the NGR coded cDNA was knocked in the Gypa, one marker of RBCs, through in vivo CRISPR‐engineering strategy. The Bio‐MVs with NGR peptides are displayed and purified from the mature erythrocytes via the specific procedure.^[^ ^54^ ^]^ Through rigorous experimental calculations, OA could be encapsulated in the functional Bio‐MVs through membrane extrusion (Bio‐MVs@OA). Besides, the shielding activity could block the accessibility of the antibody to scavenging and reducing its immunogenicity. The significant antitumor growth efficacy in subcutaneous tumors was achieved by benefiting from the excellent targeting ability of NGR modified Bio‐MVs. Compared with frequently used HEK293T for donor cells, RBCs find a balance between antitumor and antiviral immunity and have remarkable targeting ability for tumors with low immunogenicity.^[^ ^52^ ^]^ In summary, genetically modified Bio‐MVs enable efficient systemic delivery of OA, evade the host immune responses, and reduce side effects for OA therapy.

Recently, a significant number of breakthroughs have revealed pathogens, including other viruses and bacteria, have developed unique strategies such as natural tropisms and markers to enter target tissues or cells.^[^ ^55^ ^]^ Substantial efforts have been undertaken toward understanding the key features of these pathogens, and the ultimate goal is to mimic or modify them for delivering therapeutic payloads.^[^ ^56^ ^]^ The positive results of Bio‐MVs@OA also indicate that genetically engineered Bio‐MVs have real potential for encapsulating other pathogenic microorganisms such as therapeutic bacteria and other functional viruses to reduce their biotoxicity but with their biological activity maintaining.^[^ ^57^ ^]^ In addition to this, Bio‐MVs will be a favorable candidate for therapeutics of other diseases because other corresponding targeting peptides can be displayed on their surface.

#### 3.1.2 Virus Epitope Displaying for Immune Protection

Apart from the targeting and appetency with targeted cells used for OA delivery, polypeptides are also widely used in antiviral therapy, just as prophylactic vaccines.^[^ ^58^ ^]^ Commonly, an infection caused by enveloped viruses is dangerous, and designing effective vaccines is a critically important challenge.^[^ ^59^ ^]^ Most prophylactic viral vaccines are composed of attenuated or inactivated viruses because they are practical and useless as an adjuvant.^[^ ^60^ ^]^ However, the ability to revert to a pathogenic form and the high toxic potential of packed viral nucleic acids has a potential disadvantage in the clinical application process.^[^ ^58b^ ^]^ Versus attenuated or inactivated virus, the subunit vaccines (e.g., virus‐like particles, protein fragments from virus) contain no viral genetics but could reassemble the actual structure of the virus to provoke a humoral and cellular immune response.^[^ ^61^ ^]^ Nevertheless, several challenges remain in subunit vaccine development. For instance, the native conformational epitope of the viral envelop protein is difficult to produce without the post‐translational modification in eukaryotic cells.^[^ ^61c^ ^]^ In addition, the lipid membrane environment is the key to maintain a correct conformation of the multimer to reduce the possibility of producing the anti‐incorrect conformation antibody.^[^ ^62^ ^]^ Therefore, the process goal for maximizing the quantity of viron‐like epitopes against the enveloped virus is selecting appropriate strategies for viral glycoprotein anchoring and lipid membrane structure maintenance.

**Figure 2:**
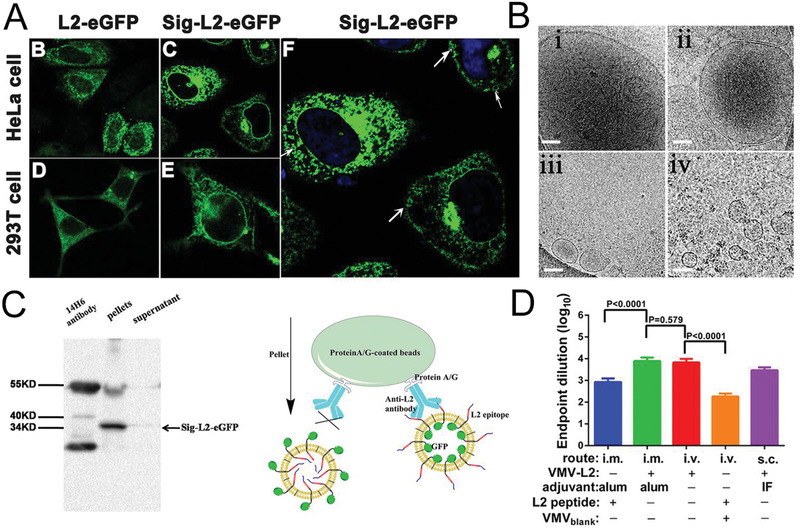
Genetically engineered Bio‐MVs with virus epitope displaying for immune protection. A) The location of recombinant protein in HeLa and HEK 293T cells with the strategy of de novo construction of recombinant membrane proteins (rMPs). B) The cryo‐EM images of purified Bio‐MVs that were incubated with different concentrations of chemical surfactants. Scale bar: 100 nm. C) Co‐IP and western blotting analysis confirmed the presence of virus epitope on the exterior of Bio‐MVs. D) Serum anti‐L2 epitope antibody titer was determined by ELISA after the mice immunized. Reproduced with permission.^[^ ^7^ ^]^ Copyright 2015, National Academy of Sciences.

To this end, Zhang et al. obtained the uniform spherical virus‐mimetic nano‐vesicles that are akin to natural viruses in size, shape, and specific immunogenicity.^[^ ^6^, ^7^ ^]^ (**Figure** **2**) First, the selected HPV L2 epitope (24 amino acids) was genetically anchoring on the HEK293T cells via the rMPs strategy mentioned before. Afterward, the Bio‐MVs that displayed HPV L2 epitope (Bio‐MVs‐L2) were produced via the appropriate chemical surfactants to accelerate the budding process. It was pleasantly surprising that vaccination of mice with Bio‐MVs‐L2 could induce a high level of antibody titers to the L2 polypeptide and elicit ≈10.5‐fold higher neutralizing antibody titers compared with the free L2 peptide group through the same immune strategy. Apart from the HPV L2 epitope, the integral membrane hemagglutinin (HA) glycoprotein (570 amino acids) from influenza A was engineered on the exterior of KEK293T cells for a specific Bio‐MVs‐HA either. HA can be anchored through its hydrophobic transmembrane region to the cell membrane, which provides a befitting lipid environment analogous to the one in enveloped viruses. The following rigorous experimental results demonstrated the Bio‐MVs‐HA have a tremendous binding capacity for neutralizing antibodies (J3F11, 13G7, 2H3, 18B10, J8A1, 16D5) that were conformation‐dependent neutralizing antibodies. That means the Bio‐MVs‐HA perfectly reproduces the structure information of HA on native virions. The great protective immune response of Bio‐MVs‐HA was also examined in vivo, just as inactivated influenza viruses. These experimental results concluded that genetically engineered Bio‐MVs are one kind of vaccine‐delivery vehicle that could elicit neutralization antibodies specific for the epitope and provide immune protection to the body as an effective vaccine. These remarkable peculiarities mainly come from the recombinant proteins produced in eukaryotic cells and are subjected to post‐translational modifications, including glycosylation acetylation, phosphorylation, and correct folding on the cell plasma membrane.

Although other kinds of natural particulates like bacterial‐derived membrane vesicles and exosomes from mammalian cells have also reported and shown excellent antigen delivery performance for membrane structure, the relative cytotoxicity of backbone proteins for Bio‐MVs and lower output of naturally secreted exosomes will hamper their further applications.^[^ ^62^ ^]^ Comparatively, genetically engineered Bio‐MVs generated from mammalian cells will successfully ease these conundrums and perform better as a virus vaccine. Specifically, recombinant viral envelope glycoproteins can anchor on the cell plasma membrane with complete post‐translational modifications like the virus infection and additional surfactants capable of controlling their size and strength.^[^ ^7^ ^]^ Therefore, the genetically engineered Bio‐MVs provide an effective, straightforward, and tunable tactic to cure a wide range of emerging enveloped viruses, in particular, the wide‐spreading and highly pathogenic viruses, including human immunodeficiency virus (HIV), influenza, and SARS‐CoV‐2 that are recently flared up and caused a global pandemic.

### 3.2 Proteins with Complex Structure Engineering

Membrane proteins that account for approximately one‐third of the proteomes of organisms have pivotal roles in virtually all biology aspects, including cell‐to‐cell signaling events, solute transport, and cellular organization.^[^ ^63^ ^]^ Their importance is underscored because they represent more than 60% of the current drug targets.^[^ ^65^ ^]^ Despite their enormous potential, few attempts have been made to gain membrane proteins widespread in biotechnology.^[^ ^64^ ^]^ The traditional dominance of strategy that membrane proteins expressed from other cells always leads to inhibition of host cellular growth, and purified protein often lacks a desired function or specificity for use in engineered processes.^[^ ^65^ ^]^ Considerable progress has been made through protein engineering and synthetic biology approaches to address these conundrums, but the results were barely satisfactory.^[^ ^22^, ^66^ ^]^ Yet, the low expression rate will directly influence further applications such as decoration for nanomedicine delivery in another aspect. Apart from these conundrums for membrane‐spanning proteins, secretory proteins such as the antibody or some viral antigens will have to meet these problems. In a way, the secretory proteins are more demanding on the stability of the protein.^[^ ^67^ ^]^ Considering the natural hydrophilic and hydrophobic properties of cell membranes for specific membrane anchoring, we presume the Bio‐MVs will be the promising alternative to overcome the difficulties of membrane proteins.^[^ ^17a^ ^]^ A genetic engineering Bio‐MVs can be nicely engineered as a surrogate for mass membrane proteins or secretory protein expression but will maintain the desired functional specificity targeting biomedical applications.^[^ ^5^, ^30^ ^]^ These include 1) the specific membrane receptor protein expression for virus neutralization; 2) the antibody expression for cancer immunotherapy and multidrug‐resistant bacterial infections (MDR); 3) multidrug‐resistant bacterial infections; 4) immune checkpoint inhibitors displaying for immunotherapy; 5) specific antigen presentation as tumor vaccine to promote anticancer immunity as well as targeted ligands anchoring for drug delivery; 6) Viral membrane proteins expression to simulate the behavior of the virus.

#### 3.2.1 Specific Receptor Displaying for Virus Capture

Apart from the designing and application of the prophylactic vaccine to avoid a pandemic of virus infection just like previously mentioned, a promising treatment to employ Bio‐MVs as a decoy to reap and detain the virus has developed either.^[^ ^68^ ^]^ The inspiration comes from the fact that the causative virus has to invade a host cell to cause infection.^[^ ^69^ ^]^ While the membrane is one of the major barriers that viruses need to conquer. The receptors expressed on the cell membrane are generally key anchoring sites for virus binding and entry, and they are also known to be important therapeutic targets.^[^ ^70^ ^]^ Bio‐MVs engineered with highly stable “mimics” of the receptor have the potential to troubleshoot the limitations of receptor‐mediated antiviral treatment with reasonable specificity and safety profiles.

**Figure 3:**
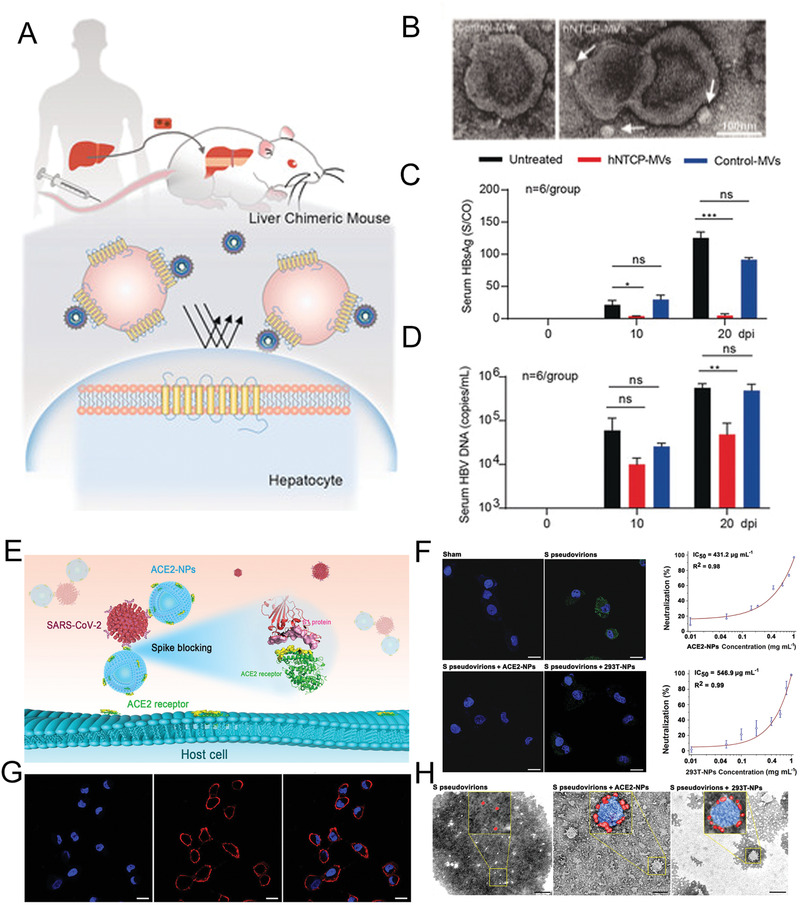
The antiviral treatment for HBV infection with genetically engineering hNTCP‐Bio‐MVs. A) Design of HBV‐infected Hu‐FRGS mice that received hNTCP‐Bio‐MVs treatment. B) TEM images of HBV‐hNTCP‐Bio‐MVs complexes negatively stained with uranyl acetate. C,D) Serum HBsAg and HBV DNA levels of untreated, Bio‐MVs treated, and hNTCP‐Bio‐MVs treated Hu‐FRGS mice with HBV infection from 0 to 20 dpi. Reproduced with permission.^[^ ^72^ ^]^ Copyright 2018, Wiley‐VCH. E) The schematic diagram of ACE2 anchoring Bio‐MVs to block SARS‐CoV‐2 infection. F) Inhibitory effect of ACE2 anchoring Bio‐MVs was evaluated via immuno‐fluorescence microscopy. S pseudovirions were traced by a FITC‐labeled antibody (green). Nuclei were stained with DAPI (blue). The scale bar is 20 µm. G) Immunofluorescence microscopy showing the location of ACE2 (red) in HEK‐293T‐ACE2 cells. Nuclei were stained with DAPI (blue). The scale bar indicates 20 µm. H) TEM images of S pseudovirions adsorbed onto NPs. The scale bar indicates 200 nm. Reproduced with permission.^[^ ^75^ ^]^ Copyright 2018, American Chemical Society.

Recently, it has been demonstrated that the pre‐S1 domain of hepatitis B virus (HBV) is a crucial determinant for HBV binding.^[^ ^71^ ^]^ As the particular receptor‐binding region of pre‐S1, the sodium taurocholate co‐transporting polypeptide (NTCP) with multiple transmembrane transporters predominantly expressed in the liver. Silencing NTCP inhibited HBV infection, while exogenous NTCP expression rendered hepatocellular carcinoma cells (HCC) susceptible to these viral infections.^[^ ^71^ ^]^ Taking inspiration from this information and genetically engineered Bio‐MVs characteristics, Liu et al. hypothesized that genetic‐engineered Bio‐MVs from NTCP‐reconstituted cells could be an effective means of binding specificity toward HBV because of their intrinsic characteristics such as virus‐mimetic shape/size and prolonged circulation time in the physiological environment.^[^ ^72^ ^]^ (**Figure** **3**A) Therefore, they successfully engineered Bio‐MVs to display hNTCP on the surface and obtained hNTCP‐Bio‐MVs as previously introduced. As shown in the experimental results, the vesicular structure, the size of hNTCP‐Bio‐MVs, and HBV‐hNTCP complexes had confirmed by transmission electron microscopy (TEM). When incubated with the HBV‐Ae/Ba stable cells in vitro, treatment with hNTCP‐Bio‐MVs rapidly reduced the HBV DNA level in the supernatant by ≈50%. Besides, hNTCP‐Bio‐MVs treatment can efficiently prevent HBV viral infection, spreading, and replication in a human‐liver chimeric mouse model of HBV infection. These positive results indicate that the antiviral effect of hNTCP‐Bio‐MVs via efficient and competitive binding to the dissociative HBV virion then subsequently blocks the viral entry process into the cells.

Apart from the HBV capture, the same design strategy has been demonstrated with the natural CD4^+^ T cell membranes for HIV inhibition and human lung epithelial type II cells or human macrophages for severe acute respiratory syndrome coronavirus 2 (SARS‐CoV‐2).^[^ ^73^ ^]^ The fabricated Bio‐MVs from CD4^+^ T cells can effectively inhibit gp120‐induced apoptosis of CD4^+^ T cells benefiting from the preserving intrinsic surface markers, including CD4 receptor and CCR5 CXCR4 co‐receptors for HIV targeting. While SARS‐CoV‐2 leverages angiotensin‐converting enzyme 2 (ACE2) expressed on the host cells as receptors for cellular entry.^[^ ^74^ ^]^ Based on this, Wang et al. successfully prepared the Bio‐MVs from ACE2‐rich cells using an extrusion method.^[^ ^75^ ^]^ (Figure 3B) This kind of Bio‐MVs contained 265 ng mg^−1^ ACE2 on the surface and acted as baits to trap the viral spike (S) protein in a dose‐dependent manner, efficiently suppressed SARS‐CoV‐2 S pseudovirions entry into host cells, and blocked viral infection in vitro and in vivo.

As a sole sharing cellular component of mammalian cells, bacteria, and viruses, the biological membrane acts as a semipermeable barrier between the cytoplasm and outside medium. Hence, the anchoring membrane proteins are often involved in basic cell behaviors such as adhesion, recognition, sensory stimuli transduction, and signal transformation. Benefiting from degeneracy and usage bias for genetic codes, we can genetically express the same functional proteins in different species, especially the membrane proteins. With the universal Bio‐MVs extraction mentioned here, almost all natural membrane proteins can bionically express on the surface to act as biomimetic nanodecoys for pathogen clearance and a pattern platform to study their interactions.^[^ ^68^, ^76^ ^]^

#### 3.2.2 Repurposing Antibody Displaying for Disease Immunotherapy

Over the past decades, the monoclonal antibodies (mAbs) have integrated the long‐sought vehicle for the targeted delivery of potent chemotherapeutic agents or as powerful mediators to manipulate anticancer immune responses.^[^ ^77^ ^]^ This excellent capacity mainly benefits from their inherent unique characteristics. First, their natural targeting and biocompatibility play a significant role in cytotoxic moieties conjugation like antibody‐drug conjugates (ADCs) for improving pharmacokinetic profiles and targeting therapeutic delivery to lesions.^[^ ^78^ ^]^ Then, their inherent capacity for invoking tumor cell death by directly blocking the specific immune checkpoints and indirect linkage to marshal the body's immune system endows their enormous power for cancer eradication.^[^ ^79^ ^]^ For example, the Fc portion of mAbs could induce antibody‐dependent cellular cytotoxicity (ADCC), complement‐mediated cytotoxicity (CMC), and antibody‐dependent cellular phagocytosis (ADCP).^[^ ^80^ ^]^ These excellent functions have strict requirements on the structure information of antibodies that directly dominate their effectiveness.

**Figure 4:**
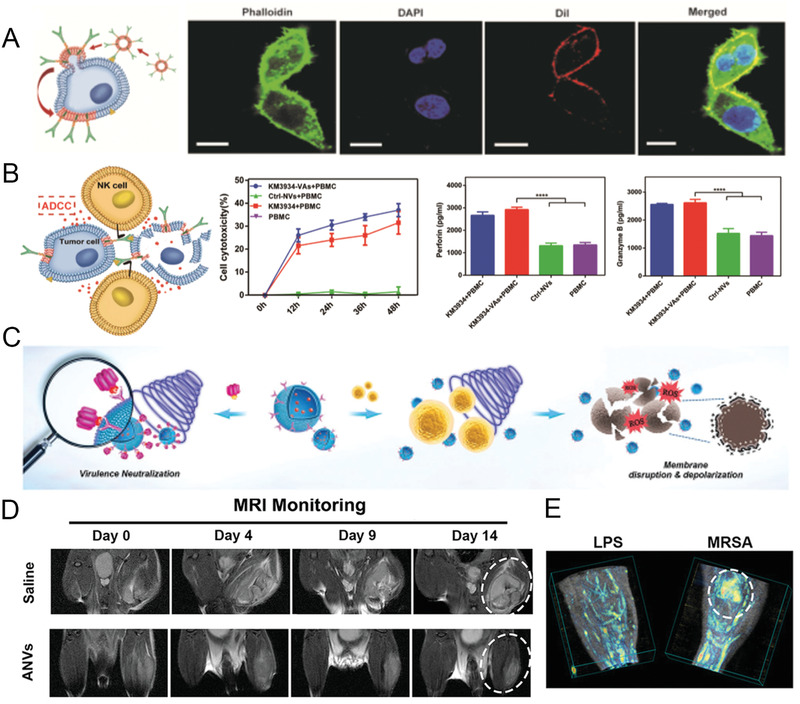
Monoclonal antibody (hGC33 and MEDI489) expressed Bio‐MVs for cancer immunotherapy and antibacterial sono‐immunotherapy. A) Demonstration of antibody‐mediated energy‐dependent membrane fusion after HepG2 cells incubated with DiI‐labeled hGC33‐Bio‐MVs. Red: Dil; Green: phalloidine. Scale bar is 20 µm. B) Antibody‐dependent cellular cytotoxicity (ADCC) cytotoxicity activity mediated by hGC33‐Bio‐MVs. Reproduced with permission.^[^ ^81^ ^]^ Copyright 2019, Wiley‐VCH. C) Schematic illustration of the antivirulence and antibacterial mechanism of Bio‐MVs with antibody MEDI4893 expressed on the surface. D) Representative magnetic resonance images (MRI) of MRSA‐infected mice after injected with MEDI489 anchoring Bio‐MVs. E) 3D photoacoustic images of MRSA and LPS infected thighs from Bio‐MVs‐treated mice. Reproduced with permission.^[^ ^42a^ ^]^ Copyright 2019, Wiley‐VCH.

Inspired by the excellent performance of Bio‐MVs for cargo delivery, Liu et al. successfully bridge Bio‐MVs and mAbs into an efficient regimen for cancer cell tracing and eradication.^[^ ^81^ ^]^ The full‐length of a monoclonal antibody (anti‐glypican 3 (GPC3) antibodies (hGC33)) was designed as the targeting moieties for HCC and genetically engineered on the exterior of cell HEK293T cell membranes.^[^ ^82^ ^]^ (**Figure** **4**A,B) Next, the specific form of Bio‐MVs with antibody displaying (vesicular antibody (VAs)) was synthesized via a particular Bio‐MVs extraction method.^[^ ^81^ ^]^ The experimental results showed complete antibody structure, functional domain orientation, and detailed conformation successfully maintained through the natural expression pathway. As expected, VAs were prepared with a stable shape and showed excellent ability for encapsulated cargo (e.g., indocyanine green (ICG), doxorubicin (Dox)) delivery. In the process, appropriate sonication powers were optimized for high drug encapsulation efficiency (>70%). More surprisingly, the encapsulated moieties were successfully internalized into the cells via antibody‐mediated energy‐dependent membrane fusion mechanism, together with the antibody transfer to the targeted cell membrane. The existence of the Fc domain on the tumor cells recruited and aggregated the NK cells into the microenvironment and mediated the ADCC for tumor necrosis. This phenomenon indicates that the genetically engineered antibody has a great capacity to guarantee structurally and morphologically stability on the cell membrane.^[^ ^19^ ^]^ As a proof of concept, such genetically engineered Bio‐MVs with specific antibody presentation can be a useful portfolio strategy for tumor‐targeted delivery of antibodies and drug molecules.

In parallel, genetically engineered Bio‐MVs with the antibody presentation were also used to combat multidrug‐resistant (MDR) bacterial infections.^[^ ^83^ ^]^ Specifically, the antibody‐based virulence factors eliminating can be used to combat immunosuppression and protect the innate immune defense.^[^ ^84^ ^]^ Thus, Pang et al. successfully prepared the Bio‐MVs with a neutralizing monoclonal antibody (MEDI4893) displaying on the membrane surface.^[^ ^42a^ ^]^ (Figure 4C,D) The selected MEDI4893 is one specific antibody against alpha‐toxin (AT) of *Staphylococcus aureus* (MRSA) in phase II trials.^[^ ^85^ ^]^ After packaging the sonosensitizer mesotetrakis (4‐sulfonatophenyl) porphyrin (TPPS) inside, this kind of Bio‐MVs could be a facile “guided missile” to bridge antibacterial sonodynamic therapy (SDT) with anti‐virulence passive immunotherapy for MDR.^[^ ^42a^ ^]^ Upon ultrasound activation, the sonosensitizers can efficiently generate ROS to kill bacteria, while the displayed antibody can assist the virulence clearance. Ultimately, the genetically engineered antibody anchoring on Bio‐MVs promises a new way for antibiotic‐free tactics to combat MDR bacterial infections robustly.

To date, apart from the cancer therapy and bacterial eradication mentioned here, the native mAbs clinical widely applies to the other kinds of diseases treatments like immune disorders and hematologic disorders, also curative efficiency affirmation.^[^ ^86^ ^]^ Despite this, single antibody‐based curing is ultimately not curative because of their limited efficiency to redundant signaling and crosstalk among different pathways within the disease microenvironment.^[^ ^19^, ^87^ ^]^ To overcome these obstacles, a broad spectrum of antibody forms are under development to enhance the clinical potential through improving their existing properties or endowing them with new functions such as a bispecific antibody, a humanized chimeric antibody, nanobody, and specifically the ADCs.^[^ ^77^, ^78^, ^87^ ^]^ In principle, all these developed forms of antibodies can be genetically engineered on the membrane surface for antibody‐decorated Bio‐MVs synthesis.^[^ ^19^ ^]^ Subsequently, purified Bio‐MVs can successfully bridge the native mAbs for immunological functions and cavity structure of Bio‐MVs for molecule loading efficiency. Undoubtedly, these ingeniously designed Bio‐MVs decorated with specific antibodies will act as an effective and generalizable alternative for ADCs to various life‐threatening diseases.

#### 3.2.3 Immune Checkpoint Inhibitors Anchoring for Cancer Immunotherapy

The immune system evolved to distinguish non‐self from self to protect the organisms from any hazardous substances.^[^ ^88^ ^]^ Because the cancer cells derived from normal somatic cells and the immune responses to dysregulate cell growth present considerable challenges. Accumulating evidence has revealed that the interaction between tumor cells and the host immune system fosters tumor immune evasion and ultimately results in tumor dissemination, relapse, and metastasis.^[^ ^89^ ^]^ Therefore, if better harnessed, the host immune system is thus a powerful tool that could significantly enhance the efficacy of cytotoxic therapy and improve outcomes for cancer patients.^[^ ^90^ ^]^ Aside from the strategy of leveraging the antibody mentioned previously, the usage of immune checkpoint inhibitors has paved the way in cancer diagnosis.^[^ ^91^ ^]^ In the process, recent breakthroughs have proved that programmed cell death protein 1 (PD‐1), and PD‐1 ligand (PD‐L1) pathway blockade is a highly promising therapeutic target and has elicited durable antitumor responses in a broad spectrum of cancers.^[^ ^92^ ^]^ Cancer tissues limit the host immune response via the upregulation of PD‐L1 and its ligation to PD‐1 on antigen‐specific CD8^+^ T cells. That means blocking the interaction between PD‐1 and PD‐L1 by antibodies or any other methods will boost the immune response against cancer cells.^[^ ^93^ ^]^ How to improve, widen, and predict the clinical response to anti‐PD‐1 therapy is a central theme in cancer immunology and immunotherapy.

**Figure 5:**
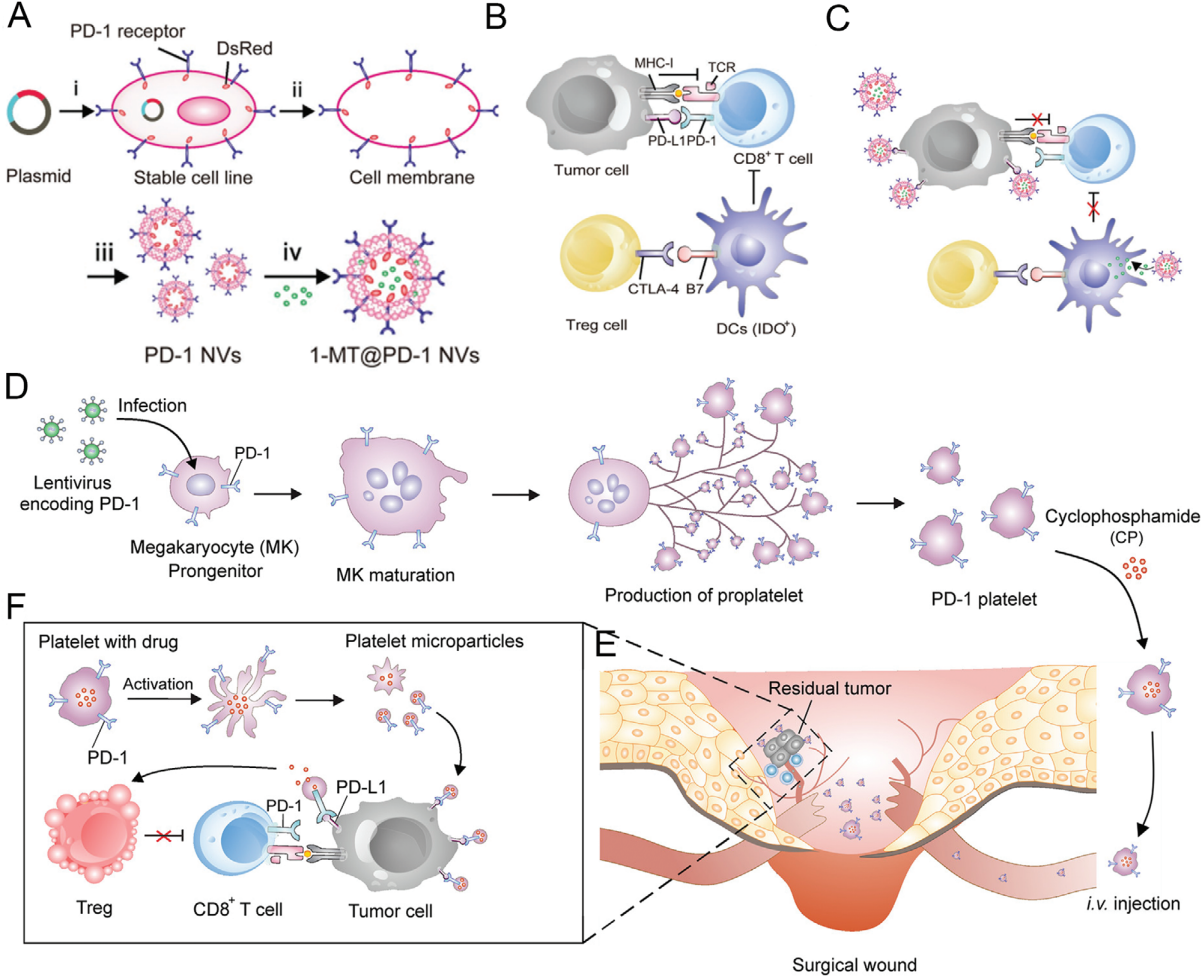
Schematic illustration and characterization of PD‐1 blockade cellular Bio‐MVs for cancer immunotherapy. A) Schematic illustration shows the preparation of PD‐1 Bio‐MVs loaded with 1‐MT. B,C) Schematic illustration shows PD‐L1 blockade by PD‐1 Bio‐MVs could revert the exhausted CD8^+^ T cells to attack tumor cells. Reproduced with permission.^[^ ^42c^ ^]^ Copyright 2018, Wiley‐VCH. D) The L8057 cell line with murine PD‐1 stably expressing and production of platelets. E) The PD‐1 expressed on the platelets can specifically concentrate on the surgery wound. F) PD‐L1 blockade by PD‐1 expressing platelets revert exhausted CD8^+^ T cells for tumor cell eradication. Reproduced with permission.^[^ ^96^ ^]^ Copyright 2018, American Chemical Society.

Considering the inherent cavity structure of Bio‐MVs for small molecules drug loading and easy functional proteins surface engineering, Zhang et al. established HEK293T cells to stably express the mouse PD‐1 receptor on the cell membrane to enhance the cancer immunotherapy through disrupting the PD‐1/PD‐L1 immune inhibitory axis.^[^ ^42c^ ^]^ (**Figure** **5**A–C) Just like the source cells, the PD‐1 preserved right side out orientation in following PD‐1 displaying Bio‐MVs, which is crucial for the effective blockade of PD‐1/PD‐L1 connection. In vitro and in vivo experimental results showed that PD‐1 anchoring Bio‐MVs intensively accumulated in the tumor sections and significantly delayed tumor growth by increasing the filtration of CD8^+^ T cells. Besides, the interior space of the Bio‐MVs can also serve as carriers for other therapeutics to perform combination delivery. Studies about indoleamine 2,3‐dioxygenase (IDO) have been widely reported, and it is an immunosuppressive molecule overexpressed by tumor and dendritic cells (IDO+DCs) to limit the proliferation and function of effector T cells.^[^ ^94^ ^]^ Therefore, with a small molecule inhibitor of IDO, 1‐methyl‐tryptophan (1‐MT) encapsulated into the PD‐1 Bio‐MVs could simultaneously block the PD‐1/PD‐L1 axis and overcome the inhibitory effects of IDO on effector T cells within the tumor microenvironment.

The common characteristic that HEK293 cells are relatively easy to be transfected highlights the novel roles for expressing exogenous genes.^[^ ^95^ ^]^ However, they are not the optimal donor cells in the strictest sense because of their immunogenicity of proteins backbone. Platelets have been widely used for designing functional nanocarriers owing to their role as circulating sentinels for vascular damage and possess a unique set of surface moieties responsible for immune evasion.^[^ ^15b^ ^]^ Unfortunately, the specific characteristics of non‐nucleated and terminally differentiated limit their clinical use because of their inability to be genetically manipulated and further modified. Considering this, Zhang et al. further developed an alternative immune‐platelet complex by genetically engineering megakaryocyte progenitor cells to express PD‐1 on the membrane and further release PD‐1 presenting platelets upon maturation.^[^ ^96^ ^]^ (Figure 5D–F) Benefiting from the genetic‐engineering method, large sources of PD‐1‐bearing platelets were obtained, and their relative immunogenicity decreased. Intriguingly, this kind of PD‐1 harboring platelets could execute the typical functions of platelet to accumulate in the surgical wound sites and block PD‐L1 on tumor cells to revert exhausted CD8^+^ T cells for eradicating residual tumor cells. Moreover, the PD‐1 platelets could be efficient cargo carriers for cyclophosphamide, an immunosuppressive drug, and improving anticancer immune response by depleting regulatory T cells (Tregs) within the tumor microenvironment. From a broad perspective, this kind of PD‐1‐bearing platelets could be ideal donor cells of Bio‐MVs preparation for further clinical nanomedicine.

Up to now, targeting immune checkpoints such as PD‐1, PD‐L1, and cytotoxic T lymphocyte antigen 4 (CTLA4) has achieved considerable benefit in multiple cancers by blocking immuno‐inhibitory signals and enabling patients to produce a significant antitumor response.^[^ ^97^ ^]^ Traditionally, the developed inhibitors to these molecules administered as single agents have resulted in durable tumor regression in some patients, and combination with other inhibitors may enhance antitumor benefit.^[^ ^98^ ^]^ Numerous additional immuno‐modulatory pathways and inhibitory factors expressed or secreted by myeloid and stromal cells in the tumor microenvironment are potential targets for synergizing with immune checkpoint blockade.^[^ ^99^ ^]^ Given the breadth of potential pharmaceutical molecules and immune checkpoint inhibitors, the genetically engineered Bio‐MVs will be the best “shell” for molecules encapsulation and functional proteins displaying. Apart from the individual inhibitor PD‐1 engineering introduced here, PD‐L1 and CTLA‐4 can simultaneously anchor the Bio‐MVs via the bioengineering method.^[^ ^100^ ^]^ This kind of dual‐targeting Bio‐MVs has excellent performance for immunosuppressive therapy in organ transplantation. Moreover, the engineered PD‐L1‐expressing platelets were also demonstrated to accumulate in the inflamed pancreas to suppress the activity of pancreas autoreactive T cells in hyperglycemic non‐obese diabetic (NOD) mice.^[^ ^101^ ^]^ Then protect the insulin‐producing *β*‐cells from destruction. Thus, the genetically engineered Bio‐MVs have the potential to bridge the preponderance of all kinds of immune checkpoint inhibitors and immunotherapy‐related cargos to be a portfolio strategy for cancer and other related disease diagnosis and therapy.

#### 3.2.4 Costimulatory Molecule Presentation to Promote Anticancer Immunity

**Figure 6:**
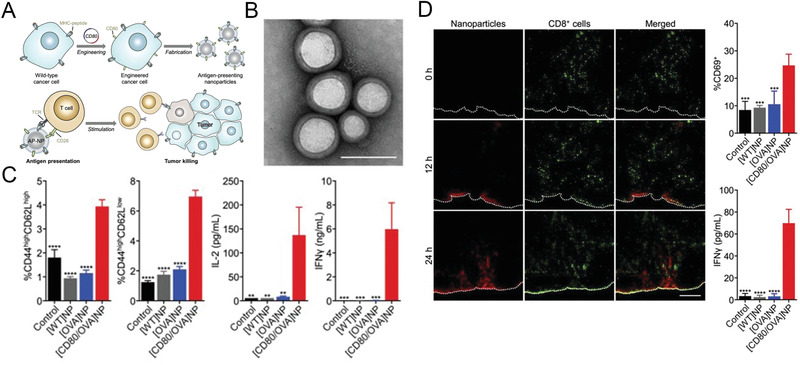
Costimulatory molecule presentation to promote anticancer immunity. A) Schematic of genetically engineered Bio‐MVs with costimulatory molecule (CD80) expressing on the surface for direct antigen presentation. B) TEM images of synthetic CD80/OVA membrane vesicles. Scale bar: 100 nm. C) After the OT‐I CD8^+^ T cells incubated with the CD80/OVA membrane vesicles for 3 days, the frequency of memory phenotypes includes CD44^high^CD62^Lhigh^ and CD44^high^CD62L^low^, as well as the secretion of IL‐2 and IFN‐Γ were monitored. D) The immunofluorescence images of draining lymph node sections from OT‐I mice after treated with CD80/OVA membrane vesicles. Red: CD80/OVA. Green: CD8^+^ cells; scale bar = 250 µm. At the same time, the expression of CD69 in the draining lymph node were monitored. Reproduced with permission.^[^ ^106^ ^]^ Copyright 2020, Wiley‐VCH.

Just as mentioned above, the tumors can utilize several immunological mechanisms in order to achieve immune escape.^[^ ^102^ ^]^ Recently emerging cancer immunotherapies address these mechanisms and mobilize the immune system in the fight against various cancers. Apart from the immune checkpoint strategies to block the immunosuppressive pathways on existing effector immune cell populations, other excellent strategies such as immune activation‐related co‐stimulatory signal delivery strategies have developed either.^[^ ^103^ ^]^ The paradigm of immune cell‐mediated antigen‐specific tumor rejection consists of three main elements: specific antigen of the tumor, efficient antigen processing or presentation, and the activation of antigen‐specific CD8+ cytotoxic T cell population.^[^ ^104^ ^]^ The greater reliance for this paradigm is correct processing and presentation of antigen by major histocompatibility complex class I (MHC I) molecules and co‐stimulatory molecules such as CD80/CD86.^[^ ^105^ ^]^ It is noteworthy that these signals are necessary to promote activation of the cognate T cells, which can target and destroy tumor cells expressing the corresponding antigen epitope. In comparison, the low efficiency of antigen presentation is a critical issue in tumor vaccine research. Jiang et al. successfully engineered a kind of Bio‐MVs from a model cancer cell line to express the co‐stimulatory molecule CD80 and enable it to present its antigens in immune‐costimulatory content with CD28 on T‐cells.^[^ ^106^ ^]^ (**Figure** **6**) They selected the B16‐F10 murine melanoma cell line as a research object because of their inherent expression of MHC I, which can present peptide epitopes from the endogenous cellular antigen.^[^ ^107^ ^]^ The cell line with co‐stimulatory molecule CD80 overexpression was successfully established via transfection with a plasmid encoding CD80 genetic sequence. The OVA was selected as a model antigen to give a wide range of immunological activity, and CD80 can engage the CD28 receptor on the T cells. Finally, functional Bio‐MVs were prepared and stabilized onto a nanoparticle substrate. It was demonstrated that Bio‐MVs formulation with CD80 expression cells could control tumor growth because this kind of biomimetic nanoformulation can directly present the model antigen to cancer‐specific T cells without the need for professional antigen‐presenting cells (APC). These strategies based on co‐stimulatory signal delivery can promote immune activation and may ultimately be used for efficient cancer therapies.

#### 3.2.5 Specific Targeting Ligand Displaying for Disease Theranostics

**Figure 7:**
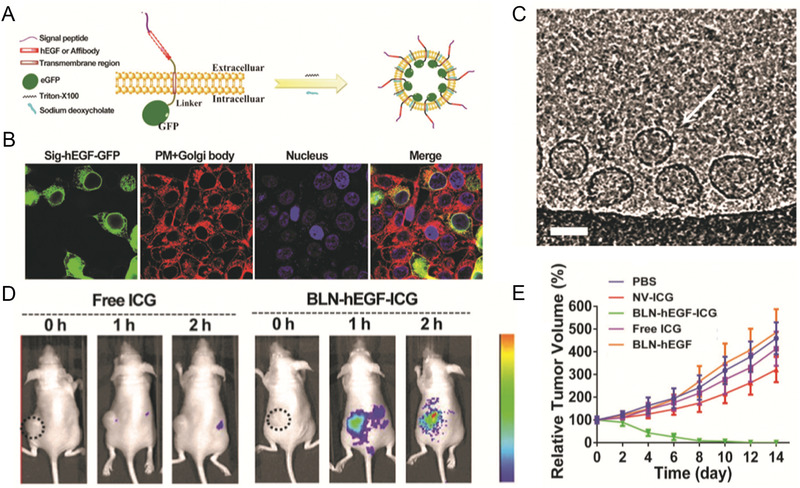
Genetic‐engineering Bio‐MVs based molecular image probes and chemotherapeutics delivery for cancer theranostics. A) Schematic of the preparation method for modified Bio‐MVs from mother cells. B) The confocal images to detecting the location of expressing hEGF‐GFP in the parent cells. C) Targeting ligand presenting Bio‐MVs (white arrows) budded from cell surface with sodium deoxycholate embedded into the plasma membrane (scale bar: 100 nm). D) In vivo FL imaging of tumors bearing nude mice after i.v. injection of Bio‐MVs‐ICG at different time points. E) Relative tumor volume changes in different treatment groups after phototherapy. Reproduced with permission.^[^ ^42b^ ^]^ Copyright 2017, Wiley‐VCH.

With the rapid development of nanotechnology and biotechnology interactions, the fusion of therapeutics and diagnostics, termed “theranostics,” has paved the way for personalized medicine.^[^ ^108^ ^]^ Specifically, the targeted ligand modification mediated active delivery tactics have promised high specificity and efficiency for theranostic capabilities.^[^ ^1b^ ^]^ Among varieties of nanocarriers, the specific liposomes greatly satisfy the criteria of systemically non‐toxic and stable in blood circulation, and liposomal Doxil has been approved for clinical use.^[^ ^109^ ^]^ Nowadays, the ligand‐functionalized liposomes also continue to thrive and have been successfully exploited in targeted delivery for further application.^[^ ^110^ ^]^ However, successfully maintaining correct spatial orientation and ligand activities after being conjugated onto the liposome surface is the major conundrum. As the most conventional liposome, like the architecture of natural particulate and easy genetically engineering with proteins anchoring, Bio‐MVs will be the potent surrogate for conventional liposomes due to excellent targeting capacities facile production. Considering these positive elements, Zhang et al. reported a biomimetic synthetic strategy to prepare Bio‐MVs that can artificially display human epidermal growth factor (hEGF) as targeting moieties for targeted drug delivery.^[^ ^42b^ ^]^ (**Figure** **7**) In brief, the functional hEGF was genetically anchored on the HEK293T cell membrane via rMPs strategy as mentioned above and then specific hEGF‐Bio‐MVs prepared through cell membrane extraction method. Compared with the natural Bio‐MVs from HEK293T or synthetic liposomes, the hEGF‐Bio‐MVs have great targeting ability to EGFR‐positive cell lines. When the photosensitized ICG was encapsulated within the hEGF‐Bio‐MVs, the synthesized Bio‐MVs have excellent efficiency for leading ICG probes to MDA‐MB‐468, just like a “guided missile.” Their targeting capability and effectiveness in vivo have been evaluated via fluorescence (FL) and photoacoustic (PA) imaging. To further demonstrating genetically engineered Bio‐MVs general applicability of targeting ligands modified “biological missile,” another HER2‐targeting ligand called Affibody was expressed on the vesicle surface.^[^ ^111^ ^]^ With the Dox encapsulated into this vehicle, both the targeting ability and therapeutic efficacy improved compared with the Doxil in vitro and in vivo. This proved that genetically modified Bio‐MVs with targeting ligands displaying could enhance molecular image probes and chemotherapeutic agents accumulating in specific receptor overexpression tumors, substantially improved the cancer theranostics, and reduced side effects.

Apart from specific ligands engineering for targeted delivery of cancer drugs and imaging moieties, other natural membrane proteins with equivalent targeting specialty can be applied. For instance, Shi et al. recently constructed the tumor necrosis factor‐related apoptosis‐inducing ligand (TRAIL) expressed cell membrane nano‐vesicles as an anti‐inflammatory platform for rheumatoid arthritis (RA) therapy.^[^ ^112^ ^]^ Due to the intricacy interactions of multiple inflammatory cytokines and the awkward drug delivery, the traditional biologic disease‐modifying antirheumatic drugs‐based treatment remains ungratified.^[^ ^113^ ^]^ In RA development, inflammatory M1 macrophages play a crucial role because they can subsequently release inflammatory cytokines and chemokines to activate or recruit other immune cells.^[^ ^114^ ^]^ Besides, M1 macrophages are the main factors causing bone erosion and cartilage destruction. It is because of the up‐regulated death receptor‐5 (DR‐5) on the M1 macrophages. The corresponding ligand TRAIL was selected for umbilical vein endothelial cell (UVEC) membrane surface engineering for M1 macrophages targeting Bio‐MVs preparation.^[^ ^112^ ^]^ Besides, another important reason for the specific choice of UVEC cells is that several inflammatory cytokines such as TNF*α* can bind its receptor on the UVEC to promote the formation of pannus, which is responsible for perpetuating RA progression. Combined with valid antirheumatic drug hydroxychloroquine (HCQ) encapsulation, these specific Bio‐MVs can largely neutralize cytokines from the synovium fluid of RA patients or the induced M1 macrophages and boost the apoptosis of M1 macrophages in vitro. Meanwhile, the specific targeting ability in arthritic joints and penetrate deeper into the inflamed paws was demonstrated via fluorescence and PA imaging. Finally, all results in vivo shown this kind of synthetic TRAIL‐engineered Bio‐MVs can effectively inhibit the progression of RA through reducing immune cell infiltration, decreasing pannus formation, and suppressing synovial hyperplasia. On account of the various proteins, including natural membrane proteins or selected protein domains such as CXCR4 and TNF*α* can be displayed on the cell membranes, it is reasonable to believe that we can customize every kind of functional Bio‐MVs for drug targeting delivery and related disease theranostics.^[^ ^16^, ^115^ ^]^

#### 3.2.6 Leveraging Viral Fusogen Anchoring for Membrane Receptors’ Transfer

**Figure 8:**
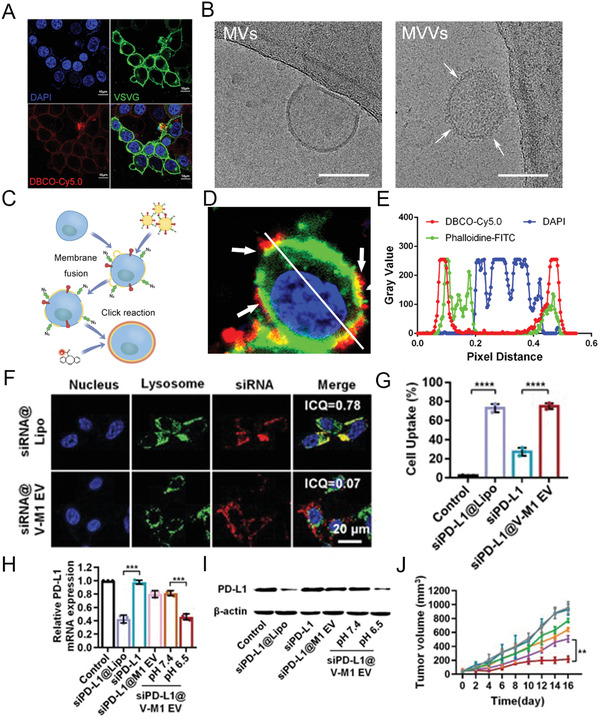
Leveraging viral fusogen anchoring for membrane receptors’ transfer and siRNA delivery. A) Confocal images for VSVG overexpressing HEK293T cells after treatment with FITC‐VSVG‐tag antibody and DBCO‐Cy5.0. DAPI (blue); FITC (green); Cy5.0 (red). Scale bar: 10 µm. B) Cryo‐EM images of Bio‐MVs that from VSVG overexpressing HEK293T cells. White arrows show the virus‐like surface spike structures. Scale bar: 100 nm. C) Schematic illustration for the fusion procedure. D,E) Verification and characterization of membrane fusion behavior via confocal microscopy in vitro. DAPI = blue; DBCO‐Cy5.0 = red. Reproduced with permission.^[^ ^120^ ^]^ Copyright 2020, Wiley‐VCH. F) Confocal observations of siRNA internalization mediated by Bio‐MVs that were genetically engineered with VSVG protein. Intensity correlation quotient (ICQ) analysis was performed to determine the colocalization. Scale bar: 20 µm. G–I) The delivering efficiency of siRNA tested with flow cytometry, RT‐qPCR, and western blot. J) Anticancer effects of siPD‐L1 encapsulating Bio‐MVs in vivo. Reproduced with permission.^[^ ^121^ ^]^ Copyright 2020, Wiley‐VCH.

As aforementioned, membrane protein defects are related to numerous human disorders that may cause problems in regulating, transport, or cellular integration of tissues.^[^ ^116^ ^]^ Hence functional protein delivery offers a potential strategy to restore the function of primitive cells.^[^ ^117^ ^]^ Despite the novel methods such as leveraging cationic nanocarriers for related proteins or gene delivery, the therapeutic vehicles with low toxicity and ideal biocompatibility are condrumns to be solved instantly.^[^ ^118^ ^]^ Considering the Bio‐MVs have various potential means to interact with the recipient cells, including endocytosis, phagocytosis, macropinocytosis, or fusing with recipient cells directly, it will have the capacity to deliver large quantities of proteins or genetic materials.^[^ ^119^ ^]^ Specifically, the membrane proteins transfer requires their reconstitution in an artificial membrane because of the immense complexity of the natural membrane. Based on this information, Ren et al. successfully fabricated one kind of Bio‐MVs via the spike VSVG engineered on the HEK293T cells.^[^ ^120^ ^]^ As shown in the cryo‐electron microscopy (cryo‐EM) images, an apparent virus‐like surface spike structure was on their surface, similar to the coronavirus. As a viral fusogen, VSVG has the inherent low‐pH activated fusogenic peculiarity with the recipient cells. Together with the azide motifs (‐N_3_) displaying on the surface, this kind of MVV can directly transfer the ‐N_3_ onto the target cells, which can mediate the following biological orthogonal reactions with dibenzocyclooctyne (DBCO)‐modified molecules. (**Figure** **8**A–E) Aside from the artificial receptor transfering for cancer diagnosis, Bio‐MVs have excellent performance in integral membrane proteins such as CD63‐GFP and glucose transporter‐4 (GLUT4‐GFP) delivery.^[^ ^27a^ ^]^ When the GLUT4 transfer to mouse muscle membranes, it will retain its functionality and allowing the increased glucose uptake of recipient cells. While electroporeting anti‐PD‐L1 siRNA into this kind of Bio‐MVs, the fusion of VSVG with cells can facilitate the direct release of iPD‐L1 into the cytoplasm and triggers robust gene silencing, leading to the efficient block of PD‐L1/PD‐1 interaction.^[^ ^121^ ^]^ (Figure 8F–J) Taking advantage of the pH‐sensitive properties of VSVG for selectively targeting the acidic environments like other focal inflammatory lesions such as rheumatoid arthritis, myocardial injury, or bacterial infection, it will be a universal strategy for membrane‐editing and exogenous proteins transferring with their intact form.

## 4 Conclusion and Perspectives

Along these lines, cellular bio‐particulates of Bio‐MVs have proven as emerging carriers for delivering a variety of functional endogenous/exogenous cargos. They offer exciting opportunities in nanomedicine because of their inherent hollow structure and more feasible engineering surface. Versus synthetic nanocarriers, the natural proteins and glycosyl groups on the membrane surface give the biocompatible Bio‐MVs prolonged and systematic retention times, enhanced permeability, limited toxicity, and better immunogenicity, as well as less reticuloendothelial system (RES) uptake. Some functional moieties anchored or encapsulated can extend the therapeutic capability of Bio‐MVs beyond their native functions and improve their multitasking ability to make them more adaptive to complex biological systems. Also, some exploratory studies have revealed that Bio‐MVs surface modification plays dynamic roles in altering the patterns and pathways of Bio‐MVs, specifically with other functional proteins modification. Succinctly, Bio‐MVs could amalgamate the preponderance of functional proteins and membrane vesicles to be excellent carriers for various drug delivery. In contrast, it is far more challenging to immobilize proteins onto Bio‐MVs but retaining the tertiary structure required for active duty. Undoubtedly, adopting a bespoke approach conjugate with the genetic‐engineering approach to Bio‐MVs modification offers the best chance of success in maintaining the conformations of amino acid sequences, sort orders, and strict specificity for their function like the cases highlighted here. Given the wealth of different functional proteins and versatile sources of cells, genetically engineered Bio‐MVs as “tailorable shells” further broaden the choice scope, design flexibility of the biomimetic systems, and apply for many diseases.

Although remarkable advantages have been validated for genetically engineered Bio‐MVs as “tailorable shells” for functional moieties delivery, many issues should be taken into account for accelerating clinical translation. For example, it is critical to ensure whether exogenous protein modifications influence the homeostasis of parent cells because each protein plays its role methodically. Although researchers have not noticed apparent signs of toxic side effects and noticeable abnormalities in Bio‐MVs treated mice, the potential toxicity and biocompatibility should be considered for the complex protein backbone and residual surfactants. Furthermore, HEK293T cells were often used as donor cells for the excellent works highlight in this progress report because they are relatively easy to be transfected and highlight the novel roles for the expression of exogenous genes. In contrast, they are not the optimal candidate because other redundant proteins may induce potential immunotoxicity. Therefore, although the excessive contamination in Bio‐MVs was virtually negligible through strict separation and careful washing, more systematic investigations are necessary: 1) selecting the autologous cell donation or patient‐derived induced pluripotent stem cells (iPSC) as donor cells for reducing immune toxicity; 2) trying to optimize the expression of the target protein and the standardized expression process of Bio‐MVs need more detailed specifications in cooperation with regulatory agencies; 3) strict criteria for quality control should be established in order to achieve reproducible engineering approaches with quality assurance. Overall, we expect the detailed introduction of genetically engineered Bio‐MVs with potential pitfalls and opportunities can facilitate deeper and broader cooperation in areas of medicine, nanotechnology, material science, bioengineering, and pharmaceutical perspectives to accelerate the clinical transformation process.

## Conflict of Interest

The authors declare no conflict of interest.
